# Nanodisc technology facilitates identification of monoclonal antibodies targeting multi-pass membrane proteins

**DOI:** 10.1038/s41598-020-58002-w

**Published:** 2020-01-24

**Authors:** Bernd Gardill, Jerry Huang, Lawrence Tu, Filip Van Petegem, Kirill Oxenoid, Christy A. Thomson

**Affiliations:** 1 Amgen Research, Biologic Discovery, Burnaby, BC Canada; 2 The University of British Columbia, Department of Biochemistry and Molecular Biology, Life Sciences Institute, Vancouver, BC Canada; 3 Present Address: Amgen Research, Munich, Germany

**Keywords:** Ion channels, Antibody therapy

## Abstract

Multi-pass membrane proteins are important targets of biologic medicines. Given the inherent difficulties in working with membrane proteins, we sought to investigate the utility of membrane scaffold protein nanodiscs as a means of solubilizing membrane proteins to aid antibody discovery. Using a model multi-pass membrane protein, we demonstrate how incorporation of a multi-pass membrane protein into nanodiscs can be used in flow cytometry to identify antigen-specific hybridoma. The use of target protein-loaded nanodiscs to sort individual hybridoma early in the screening process can reduce the time required to identify antibodies against multi-pass membrane proteins.

## Introduction

The generation of transgenic animal platforms producing human antibodies^1^ combined with the technique of hybridoma fusion^2^ has vastly aided the development of monoclonal antibodies against diverse targets^3^. Standard methods to identify monoclonal antibodies begin with the immunization of the transgenic animal with the target of interest. IgG expressing B cells from spleen, lymph node, plasma or bone marrow are then collected from the animals showing the highest antigen-specific titres and fused to an immortalized myeloma cell to generate hybridoma. Following hybridoma production, individual hybridoma clones producing antibodies specific to the target of interest need to be identified. Fluorescence-activated cell sorting (FACS) can be used to identify and isolate antigen-specific hybridoma by taking advantage of the fact that hybridoma can express the rearranged IgG from the fused individual B cell on their surface^4–7^. To do so, fluorescently labelled antibodies specific for IgG are used to isolate hybridoma successfully producing IgG. This combined with fluorescently labelled antigen allows for single cell sorting of hybridoma producing IgG specific to the antigen of interest. The single cell sorted hybridoma can then be expanded in microtiter wells and specificity of IgG secreted into the culture media can be confirmed using standard screening techniques^8^.

Because FACS of antigen-specific hybridoma relies on the addition of soluble antigen, it is generally not applicable to multi-pass membrane proteins. Instead, to identify antigen-specific hybridoma to multi-pass membrane proteins, the unsorted hybridoma are plated polyclonally with multiple hybridoma seeded per well. The IgG screened from the hybridoma culture media are polyclonal with more than one specificity. Following identification of a positive binding well, the hybridoma are then clonally plated to allow for identification of the individual hybridoma clone secreting antibodies with the properties of interest. If the immune response of the animal was poor, clones of interest may be very rare requiring plating and screening of tens of thousands of hybridoma.

As membrane proteins represent a significant proportion of drug targets^9^, we sought to develop methods for identifying hybridoma specific to membrane proteins early in the monoclonal antibody selection process. The goal thus was to reduce timelines and resources by removing the need for polyclonal plating of large hybridoma pools. However, the ability to develop and characterize the binding properties of hybridoma to multi-pass membrane proteins is hampered by the often-difficult expression and purification of these proteins in a native conformation^10^. The use of various detergents for extraction has been established and can provide an environment to solubilize proteins from the cell membrane and embed them into individual micelles^11^. Unfortunately, the solubilizing power of detergents makes them also capable of destabilizing and even unfolding membrane proteins resulting in loss of conformational epitopes. In addition, the long-term stability can be compromised, and the presence of detergents can often interfere with downstream applications (e.g. lysing of hybridoma cells during FACS).

**Figure 1:**
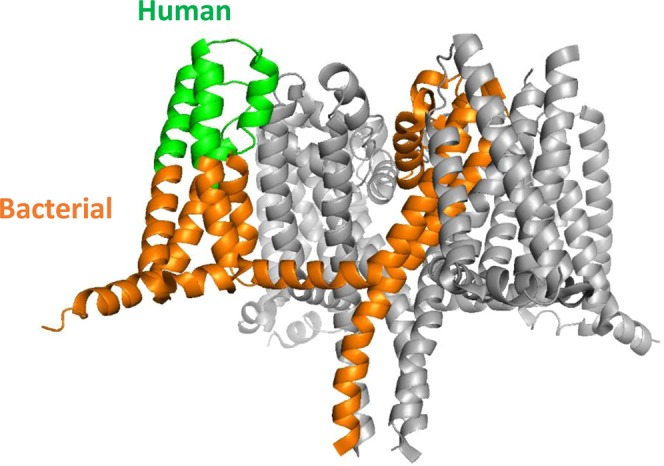
Structure of the model multi-pass membrane protein VSD4-NavAb chimera. The VSD4-NavAb chimera (PDB accession code 5EK0) is a homotetrameric protein with a central pore domain and four voltage sensing domains. Shown here is the side view of the tetramer, with one of the subunits shown in color. The sequence of the outward facing portion of the voltage sensing domain of each subunit is from VSD4 of human Nav1.7 (green) while the remainder of the sequence is that of NavAb of *Arcobacter butzleri* (orange).

Nanodisc technology offers a means to incorporate integral membrane proteins in a native-like lipid bilayer that is water soluble, making them more amenable for biochemical and biophysical characterization^12^. Nanodiscs are formed when a target membrane protein, solubilized in detergent, is mixed with an optimal ratio of phospholipid and a membrane scaffold protein (MSP)^13^. Removal of the detergent results in the target membrane protein assembling into a phospholipid bilayer held together by two molecules of MSP encircling it as a helical protein belt. In this form, the intracellular and extracellular domains of the membrane protein are exposed to solvent with the hydrophobic transmembrane domains interacting with the lipid acyl chains which in turn interact with the amphipathic helices of the MSP^14^. We sought to determine whether nanodisc technology offers a means to FACS hybridoma cells specific to target multi-pass integral membrane proteins. The chimeric VSD4-NavAb ion channel was chosen as a model multi-pass integral membrane protein system for our studies. VSD4-NavAb is a previously described homotetrameric chimeric membrane protein composed of regions of the voltage-sensing domain 4 (VSD4) of human Nav1.7 grafted onto the bacterial channel NavAb^15^. The protein assembles into a homotetramer wherein the monomeric chain consists of 6 transmembrane helices connected by 3 extracellular and 2 intracellular loops, and a cytosolic C-terminus forming a coiled coil (Fig. 1). Upon successful purification and incorporation of VSD4-NavAb into nanodiscs, we demonstrate their use in FACS of antigen-specific hybridoma. The use of target-loaded nanodiscs for FACS thus allows us to circumvent the need for polyclonal plating of hybridoma and vastly improves the efficiency of identifying antigen-specific hybridoma.

## Results

### Nanodisc production

VSD4-NavAb, a chimera of human Nav1.7 and bacterial NavAb, was chosen as a model multi-pass membrane protein for assembly into nanodiscs. Human Nav1.7 protein is a single polypeptide chain of approximately 2000 amino acids consisting of four pseudo-repeats. In contrast, bacterial homologs of Nav1.7 are homotetrameric proteins consisting of four identical subunits, each subunit containing a pore domain and a voltage sensing domain. Due to higher expression and stability, human-bacterial chimeras have been often used as surrogates for structural and biochemical studies of Nav1.7^15–17^. In this work, the constructs used follow the design of Ahuja, Mukund *et al*. 2015. Specifically, in the bacterial sequence of Nav from *Arcobacter butzleri* (NavAb), the outward facing half of the voltage sensing domain was replaced with human sequences from domain IV of human Nav1.7 channel yielding the VSD4-NavAb construct (Fig. 1).

**Figure 2:**
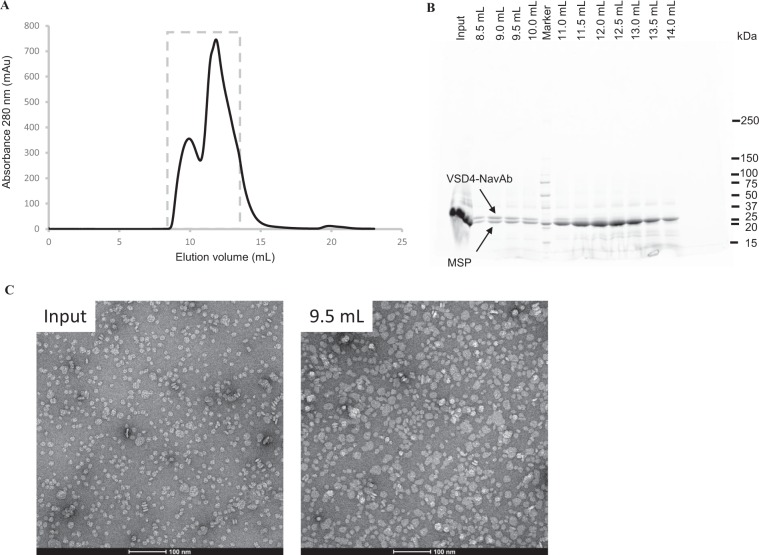
Purification and characterization of purified VSD4-NavAb nanodiscs. (**A**) Following incorporation of VSD4-NavAb into nanodiscs, size-exclusion chromatography was used to separate VSD4-NavAb-incorporated nanodiscs from empty discs. (**B**) SDS-PAGE analysis of fractions collected from SEC demonstrates that VSD4 -NavAb-incorporated nanodiscs reside in the first peak and that empty nanodiscs predominate in the second peak. (**C**) Negative stain EM demonstrating the size distribution of nanodiscs.

The VSD4-NavAb, expressed in SF9 insect cells, was solubilized in n-Dodecyl-β-D-Maltoside (DDM) detergent and purified using an N-terminal FLAG-tag. For incorporation into nanodiscs, the detergent-solubilized VSD4-NavAb was mixed with an empirically determined ratio of phospholipid and membrane scaffold protein. Size exclusion chromatography was used to separate nanodiscs containing the VSD4-NavAb from empty nanodiscs (Fig. 2A). SDS-PAGE confirmed the co-elution of the VSD4-NavAb and MSP protein indicating incorporation of the VSD4-NavAb into the nanodiscs (Fig. 2B). Negative staining electron microscopy of fractions from size exclusion chromatography was used to verify the homogeneity and size of the nanodiscs (Fig. 2C).

### VSD4-NavAb FACS

Previous immunization of VSD4-NavAb incorporated into proteoliposomes and polyclonal plating of hybridoma generated from the immunized mouse B cells, resulted in the identification of several hybridoma clones displaying antibodies that bound to the immunogen (Data not shown). The antibodies were characterized further and found to bind to either the bacterial channel portion of the chimera or the FLAG-tag and linker present in the immunogen (Data not shown). We used one such VSD4-NavAb-specific hybridoma, 141B8, to investigate whether the nanodiscs containing purified VSD4-NavAb could be bound specifically and identified by FACS. As a negative control we used a previously identified VSD2-NavAb-specific hybridoma, 141G1. VSD2-NavAb is a chimera similar to VSD4-NavAb but containing the sequences from domain II of the human Nav1.7 channel and a different linker sequence between the FLAG-tag and chimera.

**Figure 3:**
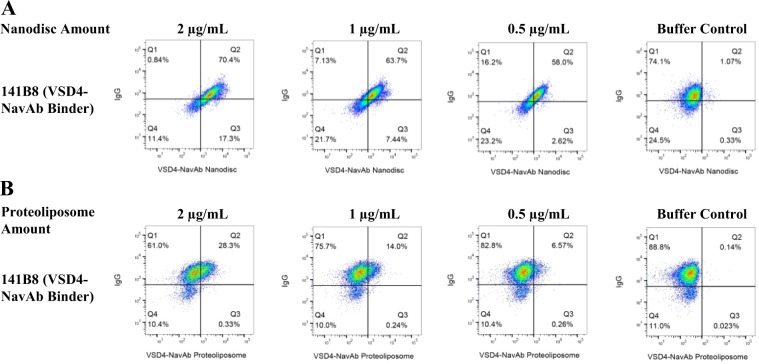
FACS comparison of VSD4-NavAb nanodiscs to VSD4-NavAb proteoliposomes in binding to hybridoma. 141B8, a hybridoma previously shown to produce antibodies that bind to VSD4-NavAb proteoliposomes, was used to investigate whether antigen-incorporated nanodiscs versus proteoliposomes could be used in FACS to identify antigen-binding hybridoma. VSD4-NavAb nanodiscs (**A**) or proteoliposomes (**B**) were biotinylated and added to the 141B8 hybridoma cells at the indicated amounts with Alexa Fluor 647-conjugated streptavidin added subsequently for detection (x-axis). Alexa Fluor 488-conjugated goat anti-mouse IgG was used to confirm surface expression of IgG on the hybridoma (y-axis). The population in quadrant 2 (Q2) represents 141B8 hybridoma expressing IgG that bind to VSD4-NavAb nanodiscs (**A**) or proteoliposomes (**B**).

**Figure 4:**
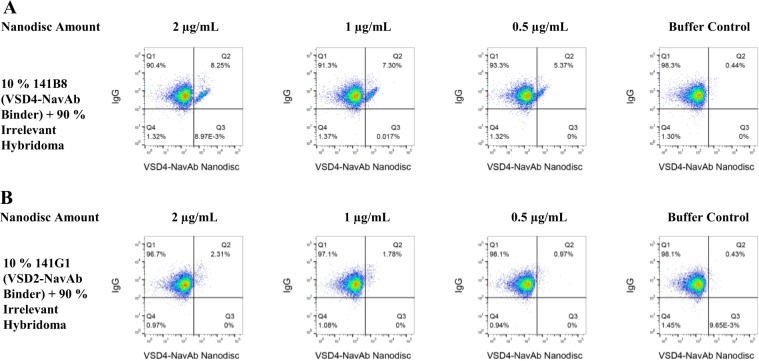
Hybridoma displaying antibody against VSD4-NavAb specifically bind to VSD4-NavAb nanodiscs. (**A**) To examine whether binding of the VSD4-NavAb nanodiscs to 141B8 was specific, a mixture containing 90% irrelevant hybridoma and 10% of the 141B8 hybridoma specific to VSD4-NavAb was examined by FACS. (**B**) To further confirm specificity, 10% of the VSD2-NavAb specific hybridoma (141G1) was likewise mixed with 90% irrelevant hybridoma and examined for VSD4-NavAb nanodisc binding. The x-axis in each plot shows binding of biotinylated VSD4-NavAb nanodiscs added to the hybridoma cells at the indicated amounts. Alexa Fluor 647-conjugated streptavidin was used for nanodisc detection. The y-axis shows display of IgG on the hybridoma surface detected by Alexa Fluor 488-conjugated goat anti-mouse IgG. Binding of the nanodiscs to a discrete population in the 141B8 hybridoma mixture is evident in Q2 whereas no binding of the VSD4-NavAb nanodiscs was observed in the 141G1 hybridoma mixture.

The VSD4-NavAb-specific hybridoma 141B8 was incubated with biotinylated nanodiscs, washed and probed for binding using fluorescently conjugated streptavidin and goat anti-mouse IgG. As shown in Fig. 3A, a distinct binding population was observed with binding increasing with increased IgG expression. For comparison, VSD4-NavAb proteoliposomes were similarly biotinylated and probed for binding to the 141B8 hybridoma (Fig. 3B). Unlike the binding observed for the nanodiscs, the positive binding population was diffuse and did not correlate as well with IgG expression. We therefore chose nanodiscs for further investigation. To examine nanodisc specificity, a hybridoma pool containing 10% of the VSD4-NavAb-specific hybridoma 141B8 was mixed with 90% irrelevant hybridoma. Biotinylated nanodiscs were incubated with the hybridoma mixture, washed and probed for binding using fluorescently conjugated streptavidin and goat anti-mouse IgG. As shown in Fig. 4A, the 141B8 VSD4-NavAb-specific population is readily identifiable in the mixture in Q2. In a control experiment, when 10% of the VSD2-NavAb-specific hybridoma 141G1 was mixed with 90% irrelevant hybridoma, no binding of the nanodiscs is evident (Fig. 4B). It was noted that the nanodiscs did not appear to be ‘sticky’ due to the lack of bulk fluorescent shift by the irrelevant population.

**Figure 5:**
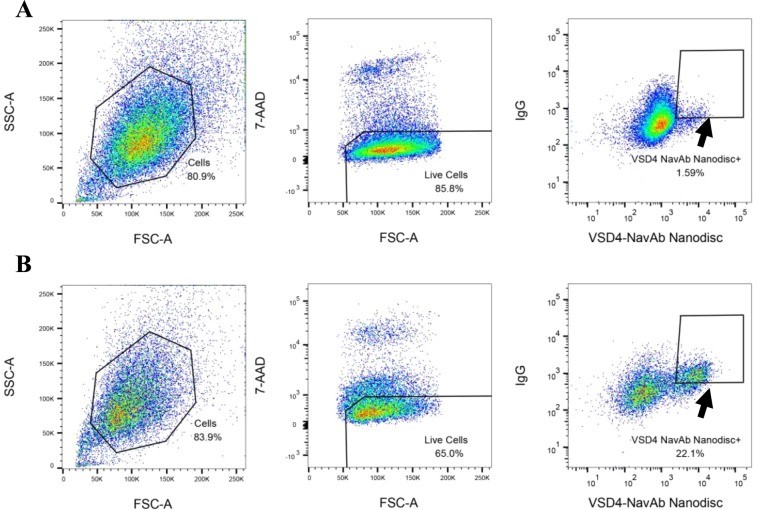
Flow cytometric sorting of VSD4-NavAb-specific hybridoma. Shown is the gating strategy used to single cell sort antigen-specific hybridoma from a hybridoma pool generated from mice immunized with VSD4-NavAb proteoliposomes. (**A**) Using SSC, FSC and 7-AAD stain to gate live cells, the live hybridoma cells were first bulk sorted based on IgG(+) expression and binding to VSD4-NavAb nanodiscs. (**B**) Following the bulk sort to enrich for antigen-specific binders, live hybridoma cells were single cell sorted based again on IgG(+) expression and binding to VSD4-NavAb nanodiscs. Alexa Fluor 647-conjugated streptavidin was used for detection of biotinylated VSD4-NavAb. IgG on the hybridoma surface was detected by Alexa Fluor 488-conjugated goat anti-mouse IgG.

We next examined whether the nanodiscs containing VSD4-NavAb could be used to identify and single cell sort hybridoma specific for VSD4-NavAb from a large polyclonal hybridoma pool. Hybridoma generated from mice previously immunized with VSD4-NavAb proteoliposomes were thawed and probed for IgG surface expression and VSD4-NavAb nanodisc binding. As shown in Fig. 5, a distinct hybridoma population was observed that both expressed surface IgG and bound to the VSD4-NavAb nanodiscs. Following a bulk enrichment step, the positive binding hybridoma were single cell sorted by FACS into eight 384-well tissue culture plates and allowed to expand over a three-week period. During this time, the hybridoma secreted monoclonal antibodies to be used in subsequent characterization assays.

### VSD4-NavAb ELISA

**Figure 6:**
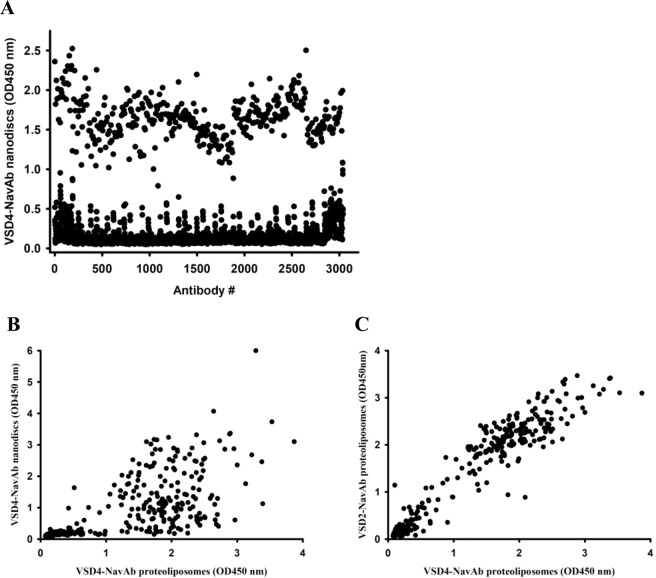
Single cell sorting of hybridoma using VSD4-NavAb nanodiscs enriches for hybridoma producing antibody to VSD4-NavAb. (**A**) Shown is an ELISA using VSD4-NavAb nanodisc coated plates to examine binding of antibodies collected in the media from the single cell sorted hybridoma. (**B**) ELISA demonstrating that antibodies shown to bind to VSD4-NavAb nanodiscs also bind to VSD4-NavAb proteoliposomes. (**C**) Antibodies that bound to VSD4-NavAb proteoliposomes bound to VSD2-NavAb proteoliposomes by ELISA.

To confirm that the single cell sorting by FACS indeed enriched for hybridoma that bound to the VSD4-NavAb nanodiscs, antibodies secreted from the individual hybridoma clones were tested for binding to VSD4-NavAb nanodiscs by ELISA (Fig. 6A). Of the 3042 wells screened, 427 bound to the VSD4-NavAb nanodiscs. (A proportion of the wells showed no binding to the VSD4-NavAb nanodiscs as expected due to less than 50% survival of our single cell sorted clones). Hybridoma clones producing binding antibodies were selected for further culture, confirmed for binding to VSD4-NavAb nanodiscs, and tested for binding to the VSD4-NavAb proteoliposome immunogen by ELISA. As shown in Fig. 6B, antibodies from the clones that bound to VSD4-NavAb nanodiscs also bound to VSD4-NavAb proteoliposomes by ELISA. The fact that the clones bound to VSD4-NavAb in both nanodisc and proteoliposome formats suggests that the binding is specific to regions found in the immunogen itself and that non-specific binding to the MSP1E3 protein forming the nanodiscs was not observed.

To assess whether the binding was specific to the the bacterial sequence of Nav from *Arcobacter butzleri* (NavAb) or the human sequences from domain IV of human Nav1.7 channel (VSD4), we compared binding to VSD4-NavAb proteoliposomes versus VSD2-NavAb proteoliposomes. Each of these proteins contain the bacterial sequence of Nav from *Arcobacter butzleri* (NavAb) but differ in that one contains domain IV and the other domain II of the human Nav1.7 channel and they each contain a different linker sequence between the FLAG-tag and chimeric protein. The mAbs were found to bind to both VSD2-NavAb and VSD4-NavAb proteoliposomes suggesting that the antibodies identified were not specific to the human sequences from domain IV of human Nav1.7 channel (VSD4) (Fig. 6C).

## Conclusions

Given the relevance of multi-pass membrane proteins as drug targets and the inherent difficulty in working with them, it is prudent to develop reagents and methods to aid in the identification of antibodies against them. Phage display with nanodiscs has been previously shown to be effective for membrane protein structural characterization and we hypothesized that they would offer a novel means of screening hybridoma panels for antigen specificity^18^. Nanodiscs were chosen over other membrane mimetic systems to test in FACS as they are thought an optimal system for displaying membrane proteins in a phospholipid environment with improved stability that resembles the native structure^14^. Indeed, using nanodiscs it has been demonstrated that specific phospholipid interactions impact native binding interactions with toxin for the TRPV1 ion channel^19^.

We chose VSD4-NavAb as a model multi-pass membrane protein for insertion into nanodiscs to represent ion channels, a difficult antibody target, and utilized previously identified hybridoma as a tool. In this work the VSD4-NavAb model multi-pass membrane protein and tool hybridoma were used to demonstrate the use of nanodiscs for cell sorting. The ability to incorporate membrane proteins into nanodiscs can be target dependent and will need to be determined empirically through screening of reconstitution conditions. If available, we recommend the use of negative stain EM to monitor whether the optimization process has yielded a homogeneous size distribution. For a review of parameters that can be taken into consideration when forming nanodiscs see Denisov and Sligar, 2017^14^.

Nanodiscs offer a means to present membrane proteins in a soluble format for FACS that more closely mimics the native protein structure. For these experiments we biotinylated the nanodisc following incorporation of the NavAb. Although labelling of target proteins is commonly used to FACS antigen-specific B-cells^20–22^, adding biotin to the antigen can result in modification of epitopes recognized by particular antibodies. As we had previously identified that the 141B8 mAb bound to NavAb, we were able to determine whether biotinylation impacted binding to its particular epitope. If control antibodies or ligand is available for a target of interest, it is recommended that binding is confirmed following nanodisc incorporation and biotinylation using ELISA. If control molecules are unavailable and epitope diversity is desired, the MSP itself can be biotinylated prior to nanodisc formation thus ameliorating the concern of adding biotin to the epitope of interest on the target protein^23^.

**Figure 7:**
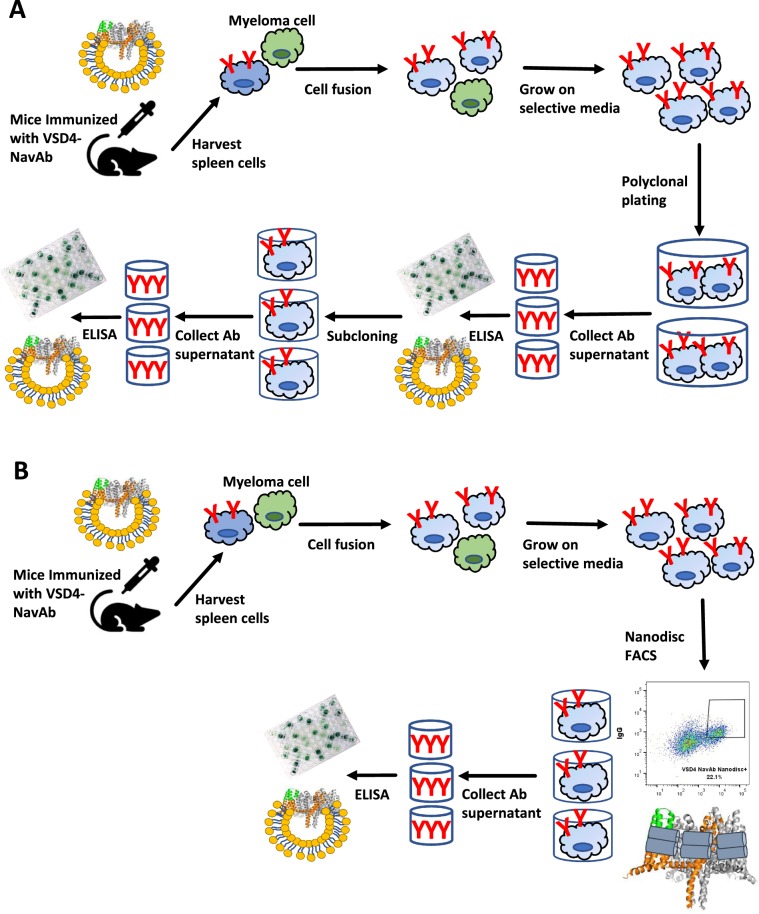
FACS of hybridoma using antigen-incorporated nanodiscs facilitates antibody discovery. (**A**) Identification of monoclonal antibodies begins with the immunization of mice with antigen, followed by isolation of immune cells. Following fusion to myeloma cells, the hybridomas are grown and secrete antibody into the media for downstream screening. In the case of multi-pass membrane protein targets, where antigen-specific hybridomas can be very rare, and when soluble FACS reagents are not available for single cell sorting, the hybridoma are plated polyclonally and screening is performed on pools of antibodies. Upon successful identification of a well of interest, the hybridoma must then be subcloned to identify the individual hybridoma generating the antibody of interest. (**B**) Incorporation of the target of interest into nanodiscs (the two helical chains of MSP are shown as grey cylinders wrapping around the NavAb; lipid molecules are omitted for clarity) enables single cell FACS of hybridoma. This allows for the identification of antigen-specific hybridoma shortly after hybridoma fusion, thus reducing timelines and resources required to screen large numbers of polyclonal plates.

We demonstrate here that incorporation of a multi-pass membrane protein into nanodisc membrane mimetics facilitates antigen-specific hybridoma sorting by FACS. The ability to positively identify antigen-specific hybridoma early in the antibody discovery process expedites antibody discovery as outlined in Fig. 7. Although preparation of validated nanodiscs containing a target multi-pass membrane protein is not trivial and requires higher amounts of starting material and time for optimization than is required for proteoliposome incorporation, the benefit of having a membrane protein in a stable form that is amenable to assays used for soluble proteins justifies the effort of nanodisc production.

## Methods

### MSP expression and purification

Expression and purification of MSP1E3 was performed following previously published methods^24^. In brief, BL21 (DE3) *E. coli* cells carrying the construct were grown at 37 °C in 2YT medium. Target protein expression was induced with 0.4 mM IPTG at OD_600nm_ 1.2. Cells were harvested after 3 hours of expression and pellets were stored at −80 °C until further processing. Cell pellets were resuspended in 50 mM Tris-HCl pH 8.0, 500 mM NaCl, 1% Triton X-100, 1 mM EDTA and lysis was conducted by ultrasonication. Insoluble particles were removed by a 30 min, 30000-g centrifugation step. The supernatant was applied to a 5 mL HisTrap column (GE Healthcare) and the column was washed with 10 column volumes (CV) 50 mM Tris-HCl pH 8.0, 500 mM NaCl, 1% Triton X-100. In a second wash step the column was flushed with 10 CV 50 mM Tris-HCl pH 8.0, 500 mM NaCl, 50 mM Cholate, 20 mM Imidazole. The target protein was eluded with a step gradient to 80% of 50 mM Tris-HCl pH 8.0, 500 mM NaCl, 500 mM Imidazole. TCEP was added to a final concentration of 2 mM and TEV protease was added for cleaving off the His-tag. Buffer was exchanged by overnight dialysis to 50 mM Tris-HCl pH 8.0, 500 mM NaCl. The His-tag and uncleaved target were then removed by a second HisTrap step where the flow-through was collected. Following a final buffer exchange to 20 mM Tris-HCl pH 7.5, 100 mM NaCl, 0.5 mM EDTA the sample was concentrated in Vivaspin concentrators (Sigma-Aldrich), flash frozen in liquid nitrogen and stored in aliquots at −80 °C.

### Membrane protein expression, purification and MSP-Nanodisc reconstitution

The VSD4-NavAb chimera was expressed and purified based on a previous publication^15^. Protein expression was conducted in SF9 insect cells, using ESF 921 medium (Expression systems) with 1x Antibiotic-Antimycotic (Gibco) and 5% FBS (Sigma-Aldrich) final concentration. Cells were harvested 48 hours after addition of virus carrying the target protein. The obtained cell pellets were stored at −80 °C until further processing. Cells were resuspended in 50 mM Tris pH 8.0, 200 mM NaCl, 0.5 mM EDTA and PMSF was added to 0.7 mM final concentration. Lysis was performed by ultrasound sonication on ice. A membrane pellet was formed by centrifugation for 30 minutes at 140000-g and 4 °C. The pellet was then homogenized and solubilized in 50 mM Tris pH 8.0, 200 mM NaCl, 1% DDM by stirring at 4 °C for 1 hour. Insoluble components were removed by an additional centrifugation step. The target protein was captured by incubation of the supernatant with anti-FLAG M2 affinity gel (Sigma-Aldrich). The beads were washed with 50 mM Tris pH 8.0, 200 mM NaCl, 0.04% DDM and protein elution was performed with 0.15 mg/mL Flag-peptide. Eluted protein concentration was measured by absorbance at 280 nm prior to nanodisc reconstitution.

To determine optimal nanodisc formation conditions, empty nanodiscs formation was first investigated by titrating MSP:lipid ratios based on previously published protocols^24^. Negative stain electron microscopy (EM) was used to determine which ratio yielded the most homogeneous size distribution. The optimal membrane scaffolding protein (MSP1E3) and lipid concentration was determined as an approximate 30-fold excess of empty nanodiscs over target protein. The lipid of choice 1,2-dimyristoyl-sn-glycero-3-phosphocholine (DMPC) was previously dissolved in chloroform, aliquoted and dried under nitrogen atmosphere. It was solubilized in 50 mM Tris pH 8.0, 200 mM NaCl, 2% DDM just before use. The reconstitution mixture of target protein, MSP and lipids was incubated at 25 °C for 1 hour before addition of BioBeads SM-2 (Biorad). Protein solution was removed from the beads after an overnight incubation. The sample was then concentrated in 30 kDa MWCO Vivaspin concentrator and injected into a Superose 6 increase 10/300 gel filtration column (GE Healthcare) equilibrated in 50 mM Tris pH 8.0, 200 mM NaCl. Fractions containing target protein loaded nanodiscs elute first and can be identified by SDS-PAGE.

### Nanodisc biotinylation

Reconstituted nanodiscs carrying the VSD4-NavAb were subjected to a biotinylation step. After a buffer exchange to 10 mM HEPES pH 7.4, 150 mM NaCl the protein was biotin labeled using EZ-Link Sulfo-NHS-LC-Biotin (ThermoFisher). Excess label was removed by buffer exchange in PD Minitrap G-25 columns (GE Healthcare). The sample was then concentrated, flash-frozen in aliquots and stored at −80 °C.

### Proteoliposome formation

Proteoliposome formation was based on published methods^25^. For reconstitution, DMPC lipid was dissolved in chloroform, dried under nitrogen and dissolved at 50 mg/ml in PBS containing 50 mg/ml sodium cholate detergent. Solution was cleared by subjecting the mixture to several freeze-thaw cycles. Lipid-detergent solution was then added to protein-detergent solution to achieve a 1:50 protein:lipid ratio (w/w) and protein concentration of 20 ug/ml. This was followed by detergent removal using BioBeads. Prior to reconstitution in liposomes, DDM-solubilized VSD4-NavAb was biotinylated using the same procedure as described for nanodiscs.

### Negative stain electron microscopy

Negative stain was performed according to standard protocols. Samples in buffer were applied to glow-discharged carbon-coated copper grids. Excess sample was removed by gently blotting the grid edge with filter paper after 40 seconds. The grid was sequentially washed in two drops of deionized water with intermittent blotting steps. The grid was then briefly dipped in a first drop of 0.75% uranyl formate, blotted, and then stained for 30 seconds in a second drop. The grid was air dried after a final blotting step. Micrographs were collected on a FEI Tecnai G2 Spirit microscope.

### Preparation of mouse hybridoma cells for Ag-Specific sorting

Fused mouse B cells were thawed and cultured in 100 mL HAT selection medium for 4 days. Cell debris from non-viable hybridomas and fusion partners were removed by pouring off the medium from the tissue culture flask. Live hybridoma cells were collected by rinsing the flask with 10 mL buffer (cold Ca + and Mg + free PBS with 2% v/v FBS) followed by gentle tapping of the flask to dislodge adherent cells. The cells were washed once in 10 mL buffer, and biotinylated VSD4-NavAb nanodiscs were added at 0.5, 1 & 2 µg/ml. The sample was left to incubate at 4 °C for 1 hour. Excess unbound nanodiscs were removed by washing with 10 mL buffer, and a cocktail containing 5 µg/mL Alexa Fluor 488-conjugated goat anti-mouse IgG (Jackson: 115-545-071) and 5 µg/mL Alexa Fluor 647-conjugated streptavidin (Jackson: 016-600-084) was added. After incubating for 30 minutes at 4 °C, the cells were washed twice with 5 mL buffer, then resuspended in 1 mL of culture medium and put through a 40 µm cell strainer. 5 µL of 7-AAD (BD Pharmingen: 559925) was added to the sample 10 minutes prior to sorting.

### Flow cytometric sorting of Ag-Specific hybridoma cells

Cells were sorted on a BD FACSAria II equipped with 488 nm laser (detection: 530/30 nm, 695/40 nm) and 633 nm laser (detection: 660/20 nm) using a 100 micron nozzle at 20 Psi sheath pressure. Ag-specific cells were enriched by gating on Alexa Fluor 488 and Alexa Fluor 647 dual fluorescent population while excluding 7-AAD fluorescent cells. An initial sort was performed using Yield precision mode to enrich target population from 1.59% to 22.1%, followed by a second sort using Purity precision mode to single cell sort target population into 384-well tissue culture plates pre-filled with culture medium. The plates were stored in tissue culture incubator (37 °C, 5% CO2), and outgrowth was assessed 7 days later by visual scoring using an inverted microscope.

### ELISA

To coat ELISA plates with VSD4-NavAb nanodiscs, 384**-**well plates (Costar 3702) were coated overnight with 10 µg/ml NeutrAvidin in PBS. Following a wash with PBS the wells were blocked with 1% milk in PBS. Biotinylated VSD4-NavAb nanodiscs were subsequently captured on the ELISA plates at 0.125 µg/ml in PBS at room temperature for 1 hour. To coat ELISA plates with proteoliposomes, passive coating was utilized. Briefly, 0.5 µg/ml VSD4-NavAb or VSD2-NavAb proteoliposomes in PBS were incubated at room temperature overnight in 384-well plates (Costar 3702). 1% milk in PBS was used to block plates. Supernatant from sorted hybridoma was diluted 1:5 in PBS with 1% milk and applied to ELISA plates for 1 hour at room temperature. Following a wash with PBS, goat anti-mouse Fc POD (Jackson 115-035-008) was added as secondary for 1 hour at room temperature. Following a wash with PBS, 1 step TMB (Neogen) was added to the wells and the reaction quenched with 1 N Hydrochloric acid after 30 minutes at room temperature. Absorbance was read on a microplate reader (Multiskan Ascent) at 450 nm.
